# Magnetic and Electrical Properties of CoRE_2_W_2_O_10_ Ceramic Materials

**DOI:** 10.1021/acsomega.3c04645

**Published:** 2023-09-28

**Authors:** Bogdan Sawicki, Elżbieta Tomaszewicz, Tadeusz Groń, Monika Oboz, Irena Gruszka, Adam Guzik, Piotr Urbanowicz

**Affiliations:** † Institute of Physics, University of Silesia in Katowice, 40-007 Katowice, Poland; ‡ Faculty of Chemical Technology and Engineering, West Pomeranian University of Technology in Szczecin, 70-310 Szczecin, Poland

## Abstract

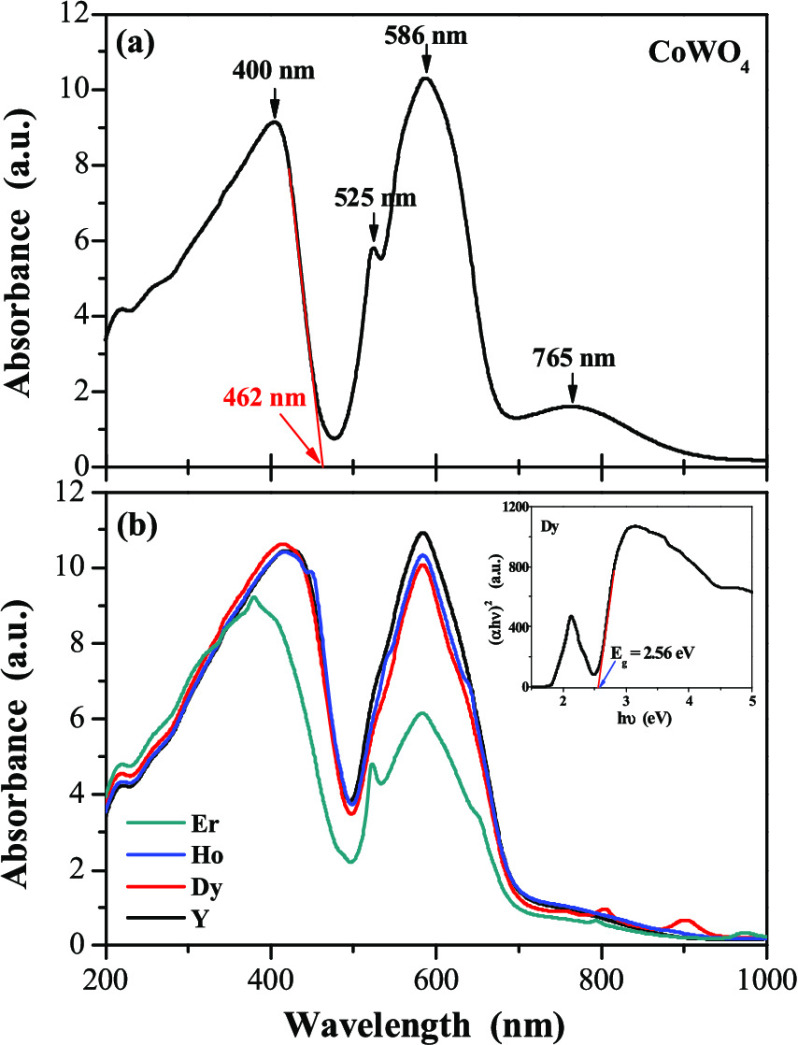

Microcrystalline samples of CoRE_2_W_2_O_10_ tungstates (RE = Y, Dy, Ho, Er) were prepared by a high-temperature solid-state reaction and then sintered into a ceramic form for unique properties and potential applications. For this purpose, structural, microscopic, ultraviolet–visible (UV–vis), magnetic, electrical, and thermoelectric measurements were performed. These studies showed a monoclinic structure, paramagnetism, short-range antiferromagnetic interactions in all samples, long-range ferrimagnetic interactions only in CoY_2_W_2_O_10_, poor *n*-type conductivity of 6.7 × 10^–7^ S/m at room temperature, strong thermal activation (*E*_a1_ = 0.7 eV) in the intrinsic region, a strong increase in the power factor (*S*^2^σ) above 300 K, a Fermi energy (*E*_F_) of 0.16 eV, and a Fermi temperature (*T*_F_) of 1800 K. The above studies suggest that anion vacancy levels, which act as doubly charged donors, and to a lesser extent, the mixed valence band of cobalt ions (Co^2+^, Co^3+^), which are located below the bottom of the conduction band and below the Fermi level, are responsible for electron transport.

## Introduction

Divalent metals tungstates, MWO_4_ (M = Mn, Co, Zn, Cd, and Pb), have been successfully used in spectroscopic and radiometric devices and as heavy and fast scintillators.^1^ Many of them, i.e., when the ionic radius of M^2+^ is relatively small, adopted a monoclinic wolframite-type structure.^2^ In turn, trivalent rare-earth (RE) metal tungstates (RE_2_WO_6_) exhibit many structural types including monoclinic symmetry with the space group *C*2/*c* (where RE = Pr–Dy).^3^ They have been used in diode-pumped crystal lasers, new-generation lighting in optical telecommunications, lidars, and other applications requiring narrow spectral sources.^4^ Our earlier studies on MPr_2_W_2_O_10_ (M = Mn, Co, and Cd) and CdRE_2_W_2_O_10_ (RE = Y, Nd, Sm, Gd–Er) tungstates obtained by high-temperature sintering of adequate MWO_4_/RE_2_WO_6_ mixtures showed that these new compounds crystallize with an orthorhombic or monoclinic structure and exhibit generally nonconductive and paramagnetic properties.^5,6^

The CoRE_2_W_2_O_10_ (CREWO) compounds, where RE = Y, Dy, Ho, and Er are isostructural, crystallize in the monoclinic system. Their lattice parameters and cell volume decrease with the decreasing radius of the RE ion. The Fourier transform infrared spectra (not shown here) suggest that the anion lattice is built by the joint WO_6_ octahedra-forming (W_2_O_9_)^6–^ groups.

We present the results of structural, microscopic, ultraviolet–visible (UV–vis), magnetic, and electrical studies of CREWO ceramic samples that have been successfully obtained by a high-temperature solid-state reaction and then sintered to a ceramic form, expecting that ceramic materials will have a wider application in electronic technology than powder ones.

## Experimental Details

**Figure 1:**
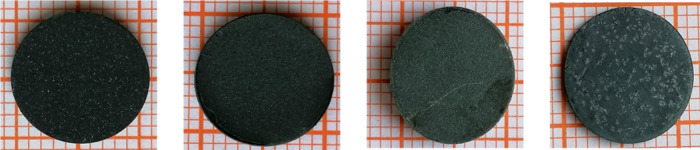
Image of the ceramic pastille after technological processing. From left to right: CoY_2_W_2_O_10_, CoDy_2_W_2_O_10_, CoHo_2_W_2_O_10_, and CoEr_2_W_2_O_10_. The length of the grille is 12 mm.

Microcrystalline samples of CoRE_2_W_2_O_10_ tungstates were obtained by a two-step synthesis. In both steps, a high-temperature solid-state reaction between appropriate reactants was applied. The following initial materials were used in the first step of synthesis: RE_2_O_3_ (99.99%, Alfa Aesar), WO_3_ (99.95%, Alfa Aesar), and CoSO_4_·7H_2_O (99.998%, Aldrich). Cobalt tungstate (CoWO_4_) and rare-earth metal tungstates (RE_2_WO_6_) were obtained analogously to our previous studies.^7,8^ In the next step, equimolar CoWO_4_/RE_2_WO_6_ mixtures were sintered in corundum crucibles, in several 12 h heating stages, in air, and at temperatures ranging from 900 to 1140 °C. After each heating period, the mixtures were slowly cooled down to ambient temperature and examined using the X-ray diffraction (XRD) method. The CoRE_2_W_2_O_10_ samples obtained in this way were pressed into pellets (5 MPa for 1 min) for magnetic and electrical studies. Next, they were crushed and ground in an agate mortar, and powders were pressed again into pellets (diameter of 12 mm) at the pressure of 19 MPa. Obtained pellets were sintered at 1150 °C for 12 h. After such technological treatment, the samples had a ceramic consistency (Figure 1).

Powder X-ray diffraction patterns of CoRE_2_W_2_O_10_ were recorded in the 10–100° 2Θ range with the scanning step of 0.013° on an EMPYREAN II diffractometer (PANalytical, The Netherlands) using Cu Kα_1,2_ radiation (λ = 1.5418 Å). The XRD patterns were analyzed by HighScore Plus 4.0 software.

UV–vis diffuse reflectance spectra of powdered samples were recorded in the spectral range 200–1000 nm using a JASCO-V670 spectrophotometer (JASCO, Italy) equipped with an integrating sphere.

**Figure 2:**
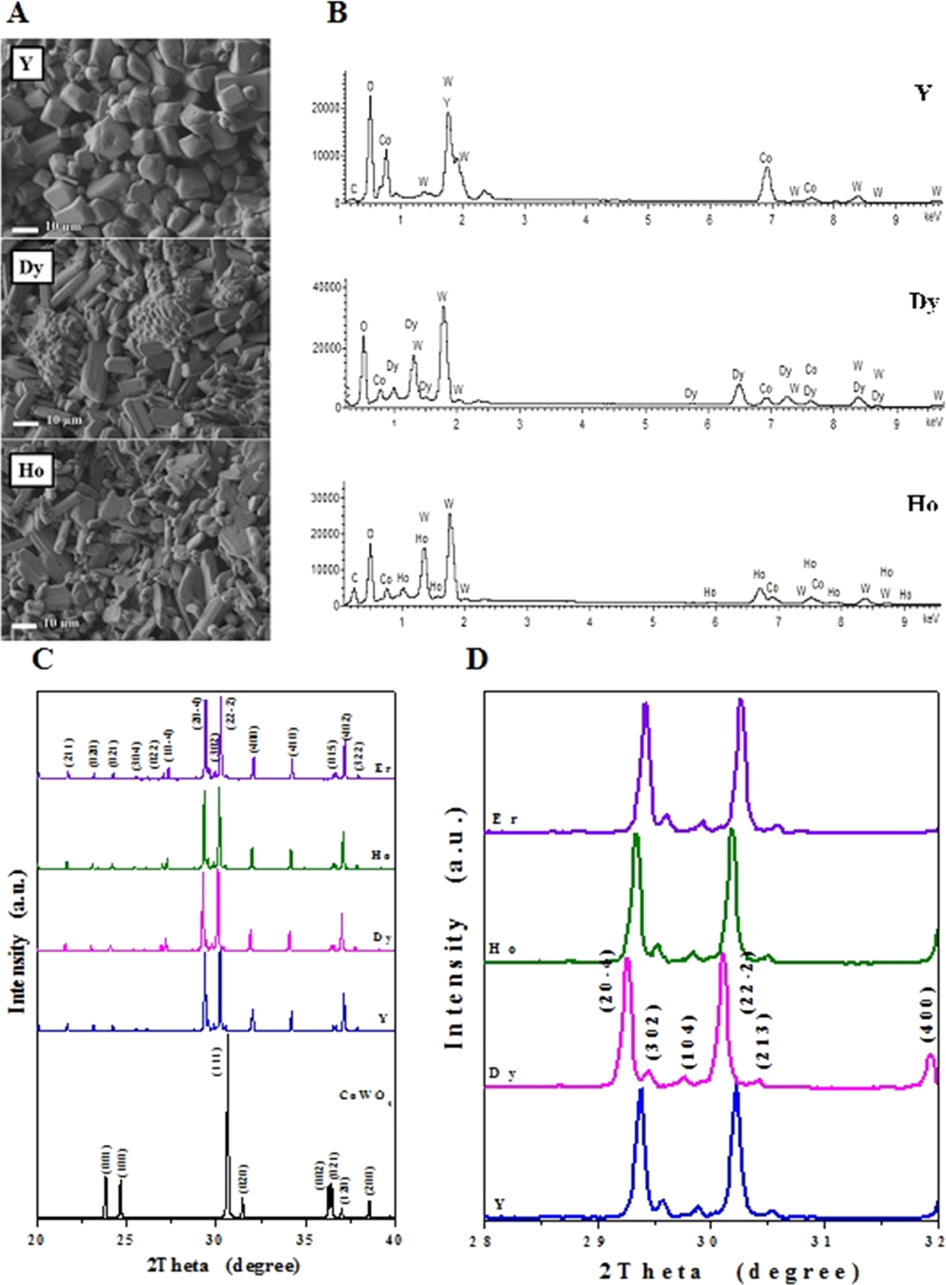
SEM images of CREWO ceramics (RE = Y, Dy, and Ho) (A); EDS spectra of CREWO ceramics (RE = Y, Dy, and Ho) (B); powder XRD patterns of CoWO_4_ and CREWO materials (RE = Y, Dy, Ho, and Er) in the range of 2Θ from 20 to 40° (C); and powder XRD patterns of CREWO materials (RE = Y, Dy, Ho, and Er) in the range of 2Θ from 28 to 32° (D).

Field-emission scanning electron microscopy (JEOL JSM 7600) was adopted to examine the morphology of the ceramics. Energy-dispersive X-ray spectroscopy (EDS) (Oxford Instrument, Abingdon, U.K.) was used for the determination of the elemental compositions of the samples. Figure 2A presents the morphology of the ceramics under study. The most frequently observed shape of the grain in the Y sample seems to resemble a truncated square pyramid with a base of about 10 × 10 μm^2^ and ∼5 μm height, whereas grains of Ho and Dy ceramics are elongated. Their dimensions seem to be measuring about 5 × 12 × 5 μm^3^. EDS mapping (Figure 2B) showed that all samples were homogeneous. The chemical compositions estimated from EDS spectra were Co_1.2_Y_2_W_1.9_O_12.4_, CoDy_1.7_W_1.9_O_10.8_, and CoHo_1.65_W_1.85_O_11.7_, respectively, very close to the nominal ones.

Magnetic susceptibility (both in ZFC and FC mode) and magnetization were measured in the temperature range of 5–300 K and in applied external fields up to 70 kOe using an MPMS-XL-7AC SQUID magnetometer (Quantum Design, San Diego, CA). The effective magnetic moment, μ_eff_, was calculated using the equation presented in refs (9,10) The effective number of Bohr magnetons, *p*_eff_, was calculated from the equation
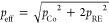
1where .^11^ To calculate *p*_eff_ from eq 1, the following *p*-values were used: 3.873 for Co^2+^, 0 for Y^3+^, 10.646 for Dy^3+^, 10.607 for Ho^3+^, and 9.581 for Er^3+^.^11^

Electrical conductivity, σ(T), of the ceramics under study was measured by the DC method using a KEITHLEY 6517B Electrometer/High Resistance Meter (Keithley Instruments, LLC, Solon, OH) and within the temperature range of 77–400 K. The sample was placed between copper electrodes and pressed mechanically. The Seebeck coefficient, *S*(*T*), was measured within the temperature range of 100–400 K with the help of a Seebeck Effect Measurement System (MMR Technologies, Inc., San Jose, CA). The electrical and thermal contact between the ceramic sample and electrodes was made by a silver lacquer mixture (Degussa Leitsilber 2000, Degussa Gold and Silber, Munich, Germany).

## Results and Discussion

### X-ray Diffraction Studies

Figure 2C shows the powder X-ray diffraction patterns of CoWO_4_ (monoclinic symmetry, wolframite-type structure, space group *P*2/*c*,^2^ JCPDS #04–007–5384) and CoRE_2_W_2_O_10_ (RE = Y, Dy, Ho and Er) in the range of 2Θ from 20 to 40°, and Figure 2D shows the powder XRD patterns of CREWO samples (RE = Y, Dy, Ho, Er) in the range of 2Θ from 28 to 32° with the most intense peaks (20–4) and (22–2). The observed diffraction lines attributed to cobalt and rare-earth metal tungstates shifted slightly toward a higher 2θ angle with decreasing RE^3+^ ion radius, i.e., in the following order: Dy^3+^→ Ho^3+^ → Y^3+^ → Er^3+^. All registered diffraction peaks were successfully indexed to the monoclinic symmetry and structure related to wolframite-type (wolframite—(Fe, Mn)WO_4_).^2^ The lattice parameters of CoRE_2_W_2_O_10_ decrease from Dy to Er.

### UV–Vis Studies

The optical energy band gap (*E*_g_) of CoWO_4_ and CREWO tungstates was determined by the method applied in our earlier studies.^12−14^ This methodology is based on a transformation of diffuse reflectance spectra into absorption ones to estimate the *E*_g_ value. When the structure of the band gap is parabolic, the absorption coefficient and optical band of materials can be determined using the Tauc relation^15,16^
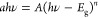
2where α is the linear absorption coefficient of a material, *h* is the Plank constant, ν is the light frequency, *A* is the proportionally coefficient characteristic of each material, and *n* is the constant associated with electron transition type.^4,5^ For materials with a direct band gap, *n* = 1/2.^15,16^

**Figure 3:**
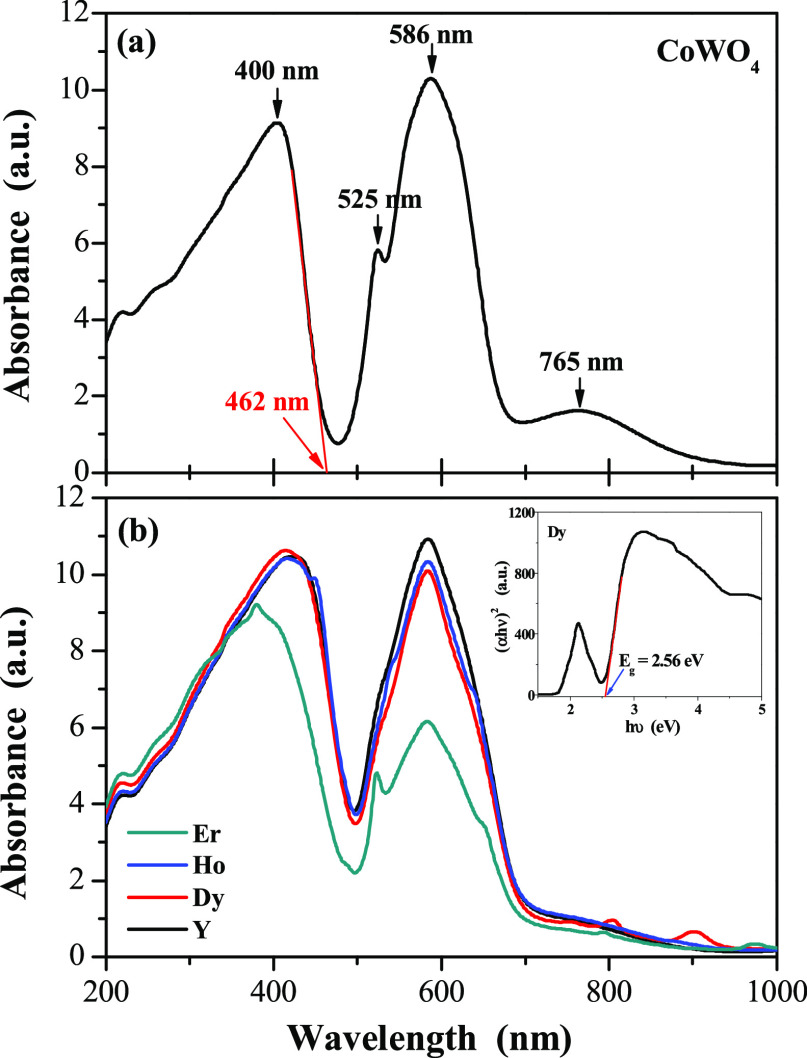
UV–vis absorption spectra of CoWO_4_ (a) and CREWO ceramics (RE = Y, Dy, Ho, Er) (b). Inset: plot of (α*h*ν)^2^ vs. photon energy (*h*ν) of the CoDy_2_W_2_O_10_ sample and determined direct band gap energy.

The UV–vis absorption spectra of CoWO_4_ and CREWO tungstates are shown in Figure 3a,b, respectively. Four absorption peaks are observed for pure CoWO_4_. The peaks with their maxima at 525, 586, and 765 nm are related to a typical forbidden *d*–*d* electronic transition coming from localized Co^2+^ ions.^17,18^ Cobalt tungstate also exhibits edge absorption at about 462 nm (broad and intense peak with the maximum at 400 nm), which is represented as a 2*p* O^2–^ → 5*d* W^6+^ transition within WO_6_ octahedra.^17,18^ The absorption spectra of CREWO ceramic materials are very similar to those recorded for cobalt tungstate in terms of the number and intensities of the observed bands. Only for the CoEr_2_W_2_O_10_ compound a shift of the absorption spectrum toward the ultraviolet spectral region is observed (Figure 3b, green line). The broad and low-intensity absorption bands ascribed to 4*f*–4*f* transitions of RE^3+^ (RE = Dy, Ho, and Er) ions in the NIR spectral range were also observed.^19−21^

The direct energy band gap, *E*_g_, of tungstates under study, obtained by extrapolating a linear part of (α*h*ν)^2^ curve of each sample to the photon energy axis (*h*ν), was found to be 2.77(1) eV (CoWO_4_), 2.54(2) eV (CoY_2_W_2_O_10_), 2.55(1) eV (CoDy_2_W_2_O_10_), 2.56(2) eV (CoHo_2_W_2_O_10_), and 2.61(2) eV (CoEr_2_W_2_O_10_). The band gap energy values of the CREWO semiconductor are clearly lower than *E*_g_ of CoWO_4_, and they increase slightly as the atomic number of the RE^3+^ ion increases.

### Magnetic Properties

**Figure 4:**
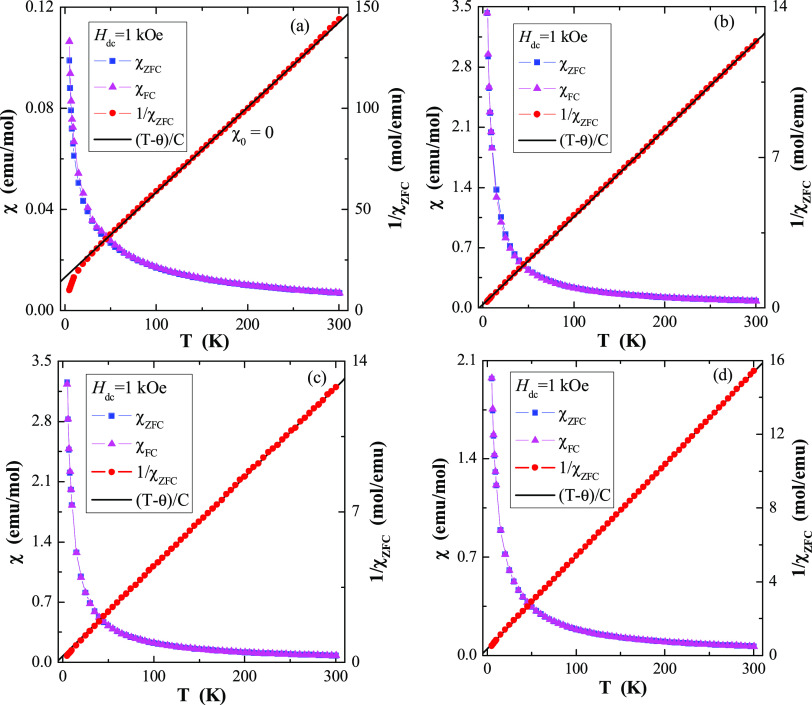
(a–d) ZFC and FC dc magnetic susceptibility χ as well as 1/χ_ZFC_ vs. temperature *T* of CoY_2_W_2_O_10_ (a), CoDy_2_W_2_O_10_ (b), CoHo_2_W_2_O_10_ (c), and CoEr_2_W_2_O_10_ (d) ceramics at *H*_dc_ = 1 kOe. The solid (black) line, (*T*-θ)/*C*, indicates Curie–Weiss behavior.

**Figure 5:**
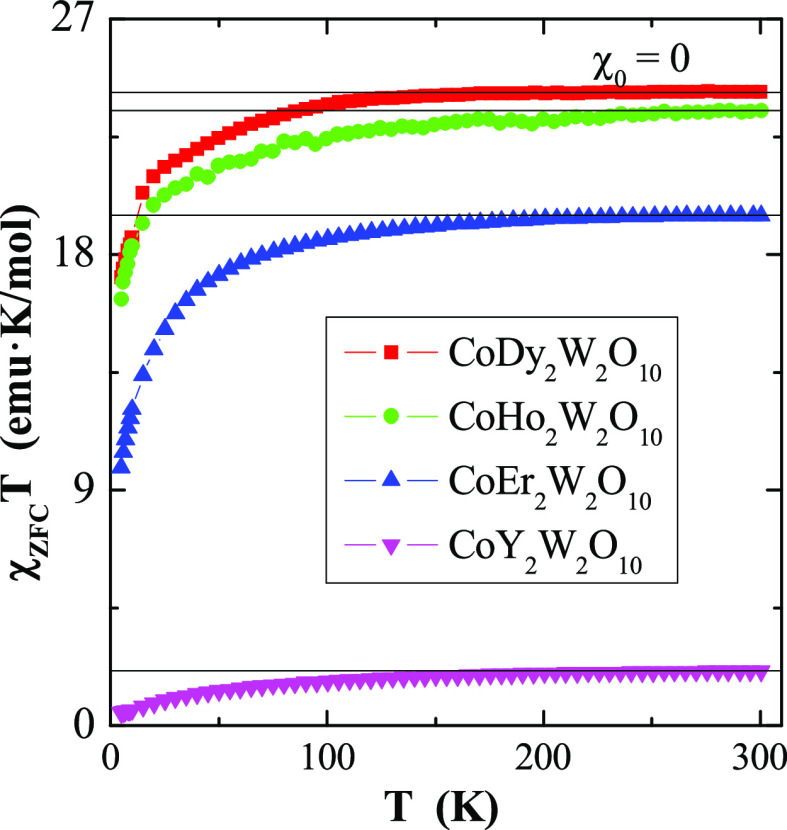
Product χ_ZFC_*T* vs. temperature *T* of CREWO ceramics. The solid line, χ*T*(*T*), indicates Curie–Weiss behavior. χ_0_ is the temperature-independent contribution of the magnetic susceptibility.

**Figure 6:**
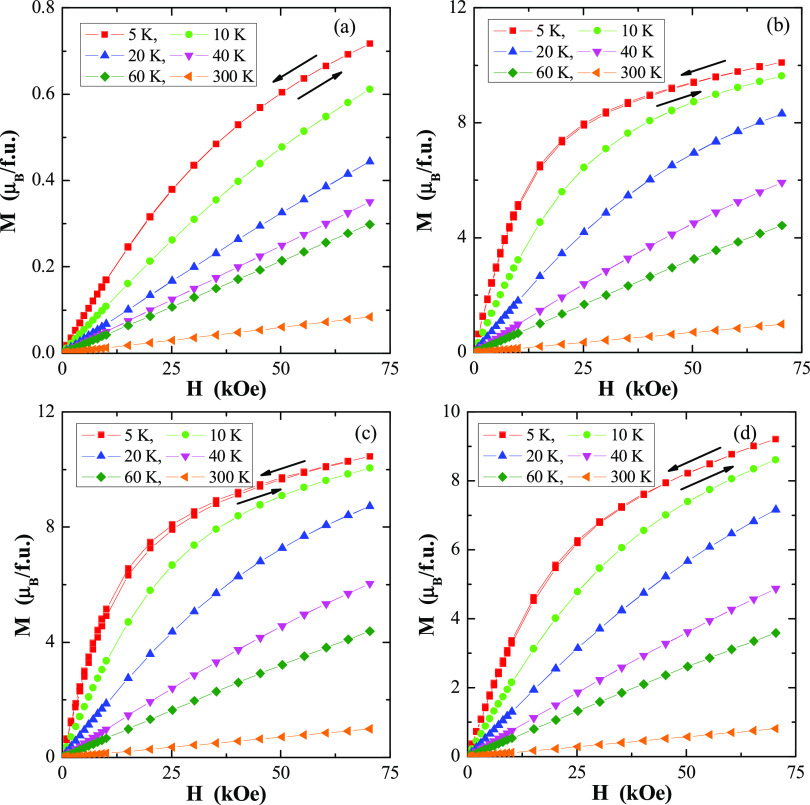
(a–d) Magnetization *M* vs. *H* of CoY_2_W_2_O_10_ (a), CoDy_2_W_2_O_10_ (b), CoHo_2_W_2_O_10_ (c), and CoEr_2_W_2_O_10_ (d) ceramics at 5, 10, 20, 40, 60, and 300 K. A run of the magnetic field is indicated by arrows.

The results of magnetic susceptibility and magnetization isotherm measurements of CREWO ceramics are depicted in Table 1 and Figures 4–6, respectively. All ceramics are paramagnetic in the temperature range of 5–300 K and have a negative value of the paramagnetic Curie–Weiss temperature of θ = −39.4 K for CoY_2_W_2_O_10_ and are ca. 6 times lower for the remaining samples (Table 1 and Figure 4), suggesting antiferromagnetic (AFM) short-range interactions and ferrimagnetic long-range ones only for CoY_2_W_2_O_10_. There was no splitting between the ZFC and FC magnetic susceptibilities for any sample, which suggests no spin frustration in the measured temperature range (Figure 4). Table 1 shows that the values of the effective magnetic moment (μ_eff_) are slightly higher than the effective number of Bohr magnetons (*p*_eff_) for CoY_2_W_2_O_10_ and slightly lower for the remaining samples. These small differences may be the result of the nonstoichiometry visible in the EDS spectra. Another reason may be the appearance of a number of Co^3+^ ions having a greater *p*_eff_ than Co^2+^ ions, while the Y^3+^ ions are diamagnetic and do not contribute to the *p*_eff_. On the other hand, in samples containing Dy^3+^, Ho^3+^, and Er^3+^ ions (where μ_eff_ < p_eff_), which are strong paramagnetics, there may be a slight deficit of Co^2+^ ions. balanced by anion vacancies acting as double donors.

**Table 1:** Magnetic Parameters of CREWO Ceramics^a^

compound	*C* (emu·K/mol)	θ (K)	μ_eff_ (μ_B_/f.u.)	*p*_eff_	*M*_0_ (μ_B_/f.u.)	χ_0_ (emu/mol)	*b* (emu·K/mol)
CoY_2_W_2_O_10_	2.382	–39.4	4.364	3.873	0.72	0	2.09
CoDy_2_W_2_O_10_	24.582	–4.07	14.021	15.546	10.10	0	24.2
CoHo_2_W_2_O_10_	24.015	–6.95	13.859	15.492	10.45	0	23.5
CoEr_2_W_2_O_10_	20.004	–7.25	12.648	14.092	9.21	0	19.5

a *C* is the Curie constant, θ is the Curie–Weiss temperature, μ_eff_ is the effective magnetic moment, *p*_eff_ is the effective number of Bohr magnetons, *M*_0_ is the magnetization at 5 K and 70 kOe, and χ_0_ and *b* are the slope and the intercept of the linear χ*T*(*T*) function, respectively.

The temperature-independent contribution of the magnetic susceptibility, χ_0_, estimated from the formula^22−24^
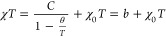
3where  is the intercept that tends to the Curie constant *C* as the temperature *T* tends to infinity and χ_0_ is the slope. χ_0_ is equal to zero for all samples, which may be the result of compensation of orbital diamagnetism and van Vleck paramagnetism (Figure 5). The intercept of the above equation *b* = 2.09 for CoY_2_W_2_O_10_ is 10 times greater for the remaining samples (Table 1).

Magnetic isotherms do not have a hysteresis, coercive field, and remanence, and they do not show saturation at 5 K and 70 kOe (Figure 6). The shape of the magnetic isotherms significantly deviates from the universal Brillouin curve. The reason for this is the small contribution of the orbital moment to the net magnetic moment and hence the stronger spin–orbit coupling. This is visible in the values of μ_eff_ and p_eff_ (Table 1), especially in the case of CoY_2_W_2_O_10_, where the magnetic moment comes only from the cobalt ions.

The elements used in the studied ceramics were selected in such a way as to emphasize their influence on the magnetic properties of the sample in the temperature range 5–300 K. A common feature of all samples is paramagnetism and short-range antiferromagnetic interactions, which are strong in the sample with the diamagnetic yttrium ion (Y^3+^) and several times weaker in samples containing paramagnetic earth ions: Dy^3+^, Ho^3+^, and Re^3+^. Closer inspection shows that in the CoY_2_W_2_O_10_ sample, at low temperatures, we find a long-range ferrimagnetic (FIM) interaction because the magnetic susceptibility deviates downward from the straight line (*T*-θ)/*C*. Replacing Y^3+^ ions with RE^3+^ ones destroys the FIM interaction and significantly weakens the AFM ones, leading to an almost perfect paramagnetic state. This means that only cobalt ions couple magnetically, while RE^3+^ ions do not because their electrons in the 4*f* orbitals are strongly screened.

### Electrical Properties

**Figure 7:**
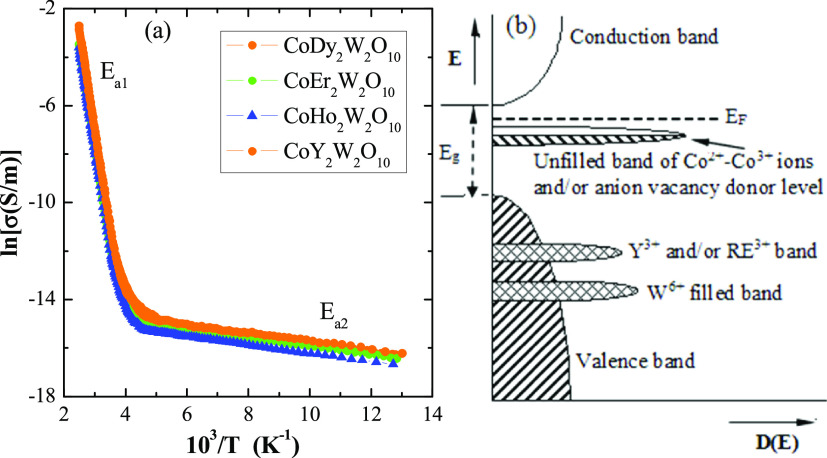
Electrical conductivity (lnσ) vs. reciprocal temperature 10^3^/T (a) and schematic representation of the electronic structure (b) of CREWO ceramics.

**Figure 8:**
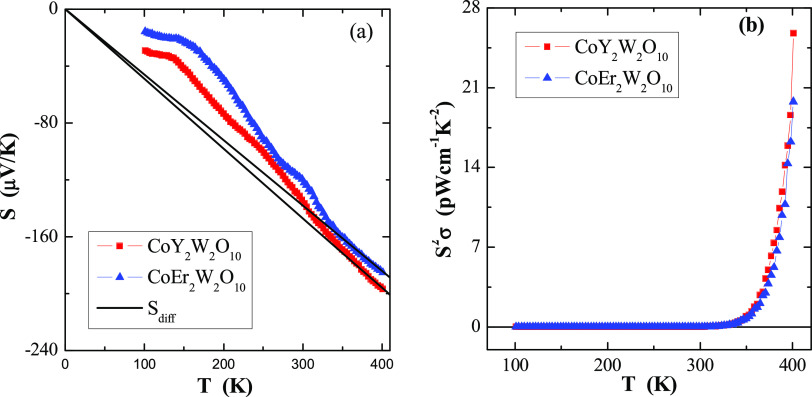
Thermoelectric power *S* (a) and power factor *S*^2^σ (b) vs. temperature *T* of CREWO ceramics. *S*_diff_ is the diffusion component of the thermopower (marked with a solid line).

The results of electrical measurements of CREWO ceramics showed *n*-type semiconducting behavior (Figures 7 and 8) of the Arrhenius type in the intrinsic temperature range of 300–400 K with the energy activation of *E*_a1_ = 0.7 eV. Next, in the extrinsic temperature range of 77–200 K, a weak electrical conductivity with the activation of *E*_a2_ = 0.02 eV was observed (Figure 7a). The value of the activation energy in the intrinsic area is almost 4 times lower than the value of the energy gap. This means that the nonzero electrical conductivity is associated with the appearance of anion vacancy donor levels in the band gap under the bottom of the conduction band and below the Fermi level *E*_F_ (Figure 7b), especially in the samples containing RE^3+^ ions. In the case of a sample containing yttrium ions, the source of *n*-type electrical conductivity may be, as suggested by magnetic studies, an unfilled band of mixed valence cobalt ions (Co^2+^, Co^3+^).

Figure 8a shows the dependence of the thermoelectric power on temperature *S*(*T*). In general, the thermopower in conventional metals is composed of two various components: a diffusion component (*S*_diff_), which is proportional to temperature according to the Mott formula at higher temperatures,^25^ and a phonon drag component (*S*_ph_), which is more complex. The *S*_ph_ contribution comes from transferring phonons at low temperatures, such as *T*^3^ below θ_D_/10, when phonons freeze out (θ_D_ is the Debye temperature), and at high temperatures, such as *T*^–1^ above approximately θ_D_/2, when the phonon’s excess momentum is limited by anharmonic phonon–phonon scattering.^26^ Typical Debye temperature for related compounds is ca. 326 K.^24,27^ The diffusion component *S*_diff_ is a direct application of the Boltzmann transport equation,^25^ described by the formula


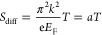
4where *e* is the elementary charge, *E*_F_ is the Fermi energy, and *a* is an empirical slope. Using eq 4, the Fermi energy, *E*_F_, can be determined by the formula
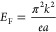
5The experimental dependence of *S*_diff_ on temperature is evident in Figure 8a, as shown by solid lines. Based on eq 5, it is possible to estimate the Fermi energy *E*_F_ and the Fermi temperature *T*_F_ (defined as *E*_F_/*k*). The values of *E*_F_ and *T*_F_ are summarized in Table 2. Compared to metals, e.g., for pure copper: *E*_F_ = 7 eV and *T*_F_ = 8.12 × 10^4^ K^28^, and to nonmetallic conductors, e.g., for Cu_1–*x*_Ga_*x*_Cr_2_Se_4_ single crystals: *E*_F_ ∼ 0.3 eV and *T*_F_ ∼ 3 × 10^3^ K,^29^ the values for ceramics under study are small. However, they are about 3 orders of magnitude higher compared to the *E*_F_ values for lead molybdate-tungstate single crystals with an admixture of Nd^3+^.^24^

**Table 2:** Electrical Parameters of CoRE_2_W_2_O_10_ (RE = Y, Er) Ceramics^a^

compound	a (μV/K^2^)	*E*_F_ (eV)	*T*_F_ (K)	*E*_a1_ (eV)	*E*_a2_ (eV)
CoY_2_W_2_O_10_	–0.49	0.15	1735	0.712	0.014
CoEr_2_W_2_O_10_	–0.46	0.16	1849	0.698	0.015

a *a* is the slope of the linear *S*_diff_(*T*) diffusion function of thermopower, *E*_F_ is the Fermi energy, *T*_F_ is the Fermi temperature, and *E*_a1_ and *E*_a2_ are the activation energies at the intrinsic and extrinsic region, respectively.

Figure 8b shows the temperature dependence of the power factor *S*^2^σ. It substantially increases with increasing temperature, i.e., in the intrinsic region above 300 K. The *S*^2^σ value of several pW cm^–1^ K^–2^ for the studied ceramics is much lower compared to the nW cm^–1^ K^–2^ value for the spinel semiconductors ZnCr_2_Se_4_:Re^30^ and to the μW cm^–1^ K^–2^ value for nonmetallic spinel conductors,^31^ but significantly higher compared to the above-mentioned single crystals, for which the *S*^2^σ value is of the order of several tens of fW cm^–1^ K^–2^.^24^

Summarizing the results of electrical investigations, it can be concluded that the ceramics under study at room temperature are characterized by low electrical conductivity σ ∼ 6.7 × 10^–7^ S/m and the value of the energy gap *E*_g_ ∼ 2.65 eV, i.e., close to the semiconductor–insulator transition. If a large share of ionic bonding is taken into account in such compounds, nonzero electrical conductivity is mainly due to vacancies and structural defects described in the literature,^32−34^ and to a lesser extent to the mixed valence band of cations.^35,36^ In the studied ceramics, the (Co^2+^, Co^3+^) band is unfilled, and oxygen vacancies act as doubly charged donors, which is illustrated by a schematic representation of the electronic structure (Figure 8b). The *E*_F_ and *E*_a1_ values indicate that both the mixed valence band and the vacant donor level are deep in the energy gap.

## Conclusions

CREWO ceramics obtained by a high-temperature solid-state reaction and then sintered into a ceramic form were characterized by structural, microscopic, UV–vis, magnetic, electrical, and thermoelectric power measurements. These studies have shown that all ceramics crystallize in a monoclinic structure, are homogeneous, and have a composition close to the nominal one. A common feature of all samples is paramagnetism, which comes from RE^3+^ and Co^2+^ ions, and short-range antiferromagnetic interactions, which come from Co^2+^ ions only. The confirmation of the latter is the appearance of long-range ferrimagnetic interactions only in the CoY_2_W_2_O_10_ sample, where Y^3+^ is a diamagnetic ion and does not contribute to the effective moment. In turn, strong paramagnetic RE^3+^ ions suppress the AFM interactions and destroy the FIM ones. The consequence of this is the appearance of a band of mixed valence of cobalt ions and vacancy donor levels.
